# Dynamic evaluation method for planning sustainable landfills using GIS and multi-criteria in areas of urban sprawl with land-use conflicts

**DOI:** 10.1371/journal.pone.0254441

**Published:** 2021-08-27

**Authors:** Chelsea Langa, Junko Hara, Jiajie Wang, Kengo Nakamura, Noriaki Watanabe, Takeshi Komai

**Affiliations:** 1 Graduate School of Environmental Studies, Tohoku University, Sendai, Miyagi, Japan; 2 Research Institute for Geo-Resources and Environment, National Institute of Advanced Industrial Science and Technology, Tsukuba, Ibaraki, Japan; Gebze Teknik Universitesi, TURKEY

## Abstract

Landfill site selection is problematic in many countries, especially developing nations where there is rapid population growth, which leads to high levels of inadequate waste disposal. To find sustainable landfill sites in sprawling cities, this study presents an approach that combines geographic information system (GIS) with multi-criteria (social, environmental and, technical criteria) and the population growth projection. The greater Maputo area in Mozambique was selected as a representative city for the study, which is undergoing rapid urbanization. Six criteria, i.e., land use, transport networks, hydrology, conservation reserve, geology and slope, were considered and overlaid in the GIS using an analytic hierarchy process (AHP). The arithmetic projection of the population trend suggests that the greater Maputo area is experiencing a rapid and uncontrolled population growth, especially in Matola city. These pronounced changes in population then significantly change the landfill placement decision making. Dynamic and static scenarios were created, based on the analysis of multi-criteria and the areas likely to undergo future increased population growth. A comparative evaluation in a scenario of dynamic behavior considering future population showed that suitable areas for landfill sites have been drastically modified due to social and environmental factors affected by population distribution in some regions. The results indicate that some suitable areas can generate land use conflicts due to population growth with unplanned land use expansion. Finally, the western part of Matola city is recognized as the most recommendable landfill site, which can serve both Maputo and Matola city with affordable costs. This study provides an effective landfill placement decision making approach, which is possible to be applied anywhere, especially in developing countries to improve sustainable municipal solid waste management systems.

## Introduction

Interest in identifying optimal landfill sites has been growing due to the importance of these facilities in a sustainable municipal solid waste management systems (MSWM). There are various types of effective landfills and about 75.5% of the world’s solid waste is disposed of in such facilities [1]. However, developing countries are still challenged with treatment and disposal of waste due to the growing quantities resulting from rapid population growth and lifestyle improvement [2,3].

At present, developing countries are still dumping about 33% of the global municipal solid waste (MSW) in open spaces, without previous separation and treatment [1]. For instance, in many southern African cities, the combination of uncontrolled urban expansion with population growth has resulted in the prolonged use of existing landfills and the creation of illegal dumping sites [4–6]. This type of disposal leads to environmental degradation and life quality deterioration by contaminating soils, surface and groundwater degradation, as well as the atmosphere with chemicals and organic pollutants [4,7,8]. Moreover, as cities and their population grow rapidly, land-use conflicts may also arise during landfill placement because landfill sites tend to be undesirable facilities [9,10]. It is more urgent than ever to improve the waste management services such as collection, transportation, and disposal in developing countries.

A sustainable landfill site should cause minimum environmental disturbance, have least financial costs possible, cause no harm to human health and well-being, and be accepted by citizens [11–13]. Nonetheless, meeting these goals can be challenging in developing countries due to rapid urbanization and increase in waste generation [2,3,12,14].

Many environmental scientists and other research fields have used Geographic Information Systems (GIS) to select landfills due to the ease with which vast amounts of data can be handled, stored and analyzed, in combination with multi-criteria decision methods (MCDM) to achieve optimum site selection [15–21]. [22] used a GIS approach that employed the analytic hierarchy process (AHP) in Turkey to analyze and combine general criteria; [23] used GIS with fuzzy AHP-based multi-attribute decision making to develop a method for precise site selection in Iran; [24] developed a site selection methodology based on GIS and AHP focused on minimizing waste of land in highly urbanized and developed cities.

However, despite these advances in landfill site selection, studies examining population growth and its effects on the placement of waste infrastructure are still limited. As cities and their population growth, land-use conflicts may arise because waste sites tend to be undesirable facilities [10].

In Maputo city, the capital of Mozambique, the citizens have been using landfill sites for about 48 years, but all of these sites are facing failure. The city has experienced uncontrolled urbanization, in the last twenty years, and the types of waste being disposed are changing along with improvements in lifestyle [5,14]. Waste planners have been experiencing conflicts with citizens, when selecting new landfill sites due overlapping of housing areas with land earmarked for landfilling [5,25].

The main goal of this study is to present a methodology for landfill placement in sprawling cities using GIS tools combining social, environmental, technical criteria and population growth projection. To achieve this goal, future population will first be projected, then, a scenario considering the projected future population will be created; finally, by comparing the current and future scenarios, conflict-free areas can be identified for landfills.

## Materials and methods

### Description of the study area

**Fig 1:**
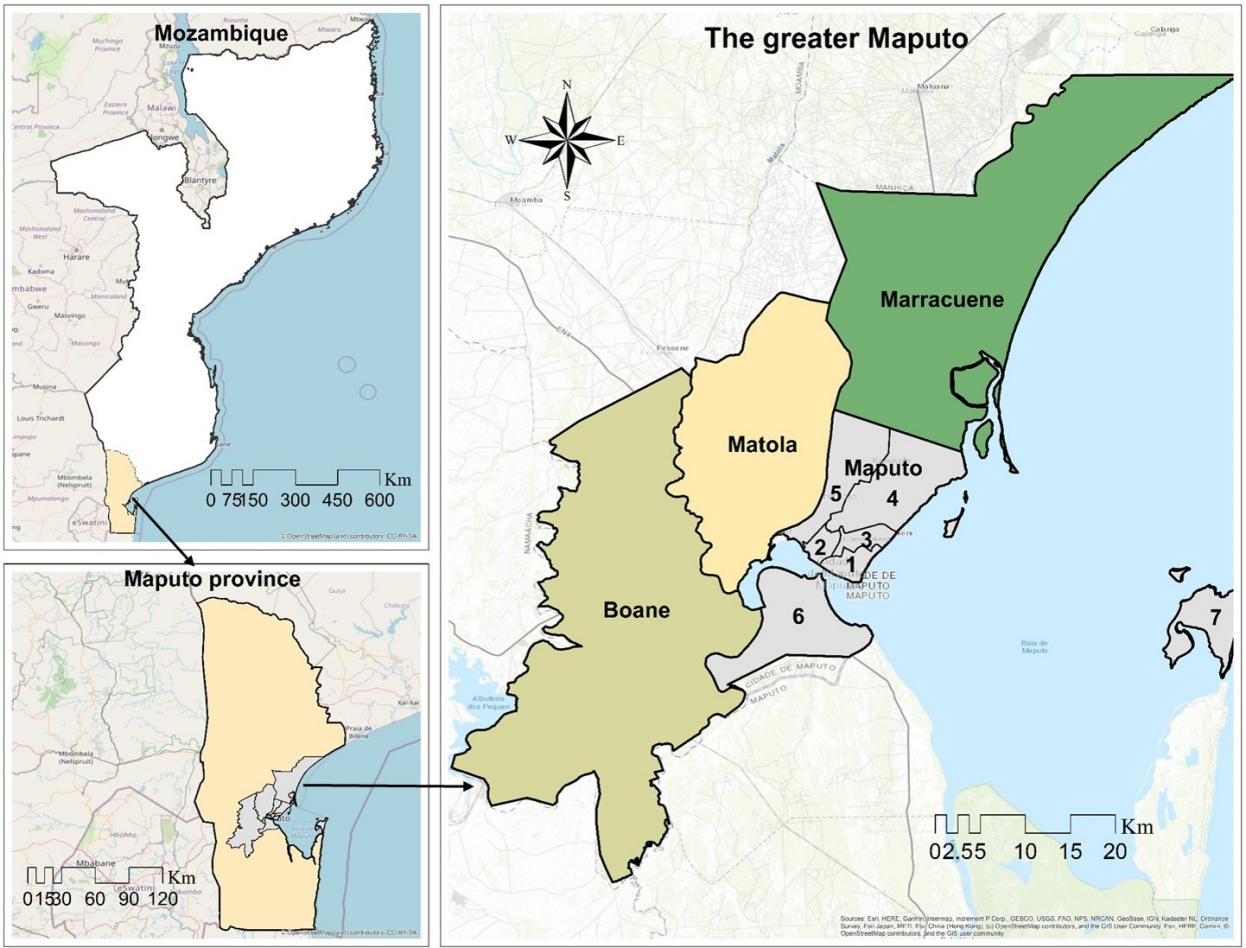
The location of the greater Maputo region. The base maps (World topographic map) were obtained at ArcGIS Online and are the property of ESRI, used herein under license [28]. The copyrights belong to ESRI, but according to the terms of use, the copyright holder does not need to apply for permission to use because it is free for academic publications, and can be used freely and commercially under the CC BY 4.0 license.

The greater Maputo region in southern Mozambique (Fig 1) occupies an area of approximately 2,000 km^2^ and is under the jurisdiction of four municipalities: Maputo, Matola, Boane, and Marracuene, all located in the south of the country along the coast. The climate of the area is tropical savannah, with a wet and warm season from October to March, and a cool and dry season from April to September [26,27].

Maputo and Matola are categorized as cities, whereas Boane and Marracuene are villages [29]. Maputo city is also the capital city of the country and is further composed of seven major administrative areas, namely boroughs 1 to 7 [30] (Fig 1). However, for this study, borough 7 is not considered due to being separated from the mainland.

**Fig 2:**
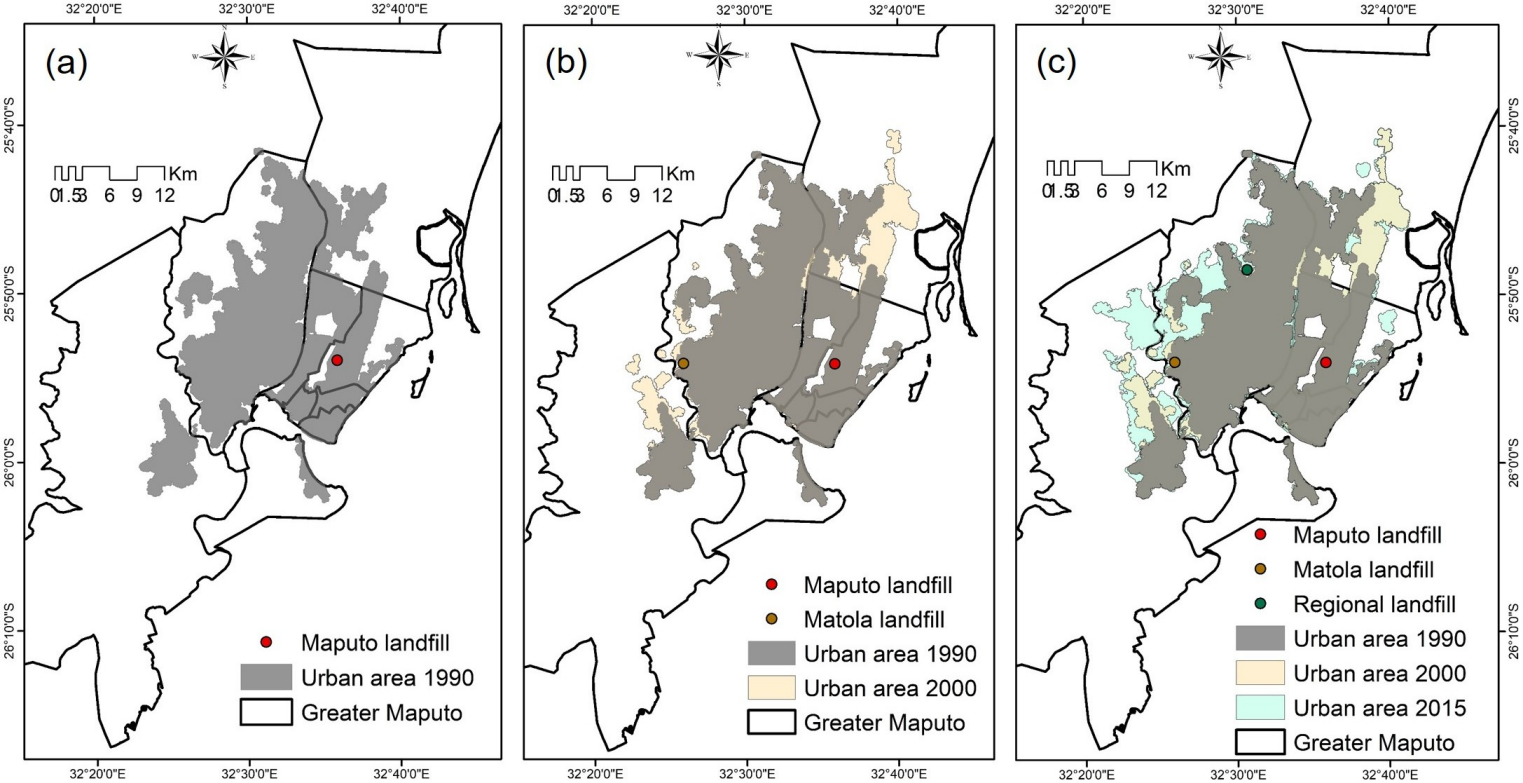
Urban expansion tendency. (a) year 1990; (b) year 2000; (c) year 2015. Maps used in Fig 2 were edited from Landsat [45] and USGS EROS [46].

The total population of the greater Maputo area has expanded from approximately 1 million inhabitants in the 1990 decade to approximately 3 million people in 2017 (Fig 2) [29], where Maputo city supports approximately 54% of this population with the population density closer to 3600 people/km^2^ in 2017. Due to inadequate institutional coordination and poor space planning, Maputo city is experiencing uncontrolled urban sprawl to neighboring cities, especially Matola city (Fig 2), [29]. This rapid expansion resulted in the increase in the demands for waste collection and disposal, and brings severe challenges in MSWM [5,25].

### Spatial analysis

**Fig 3:**
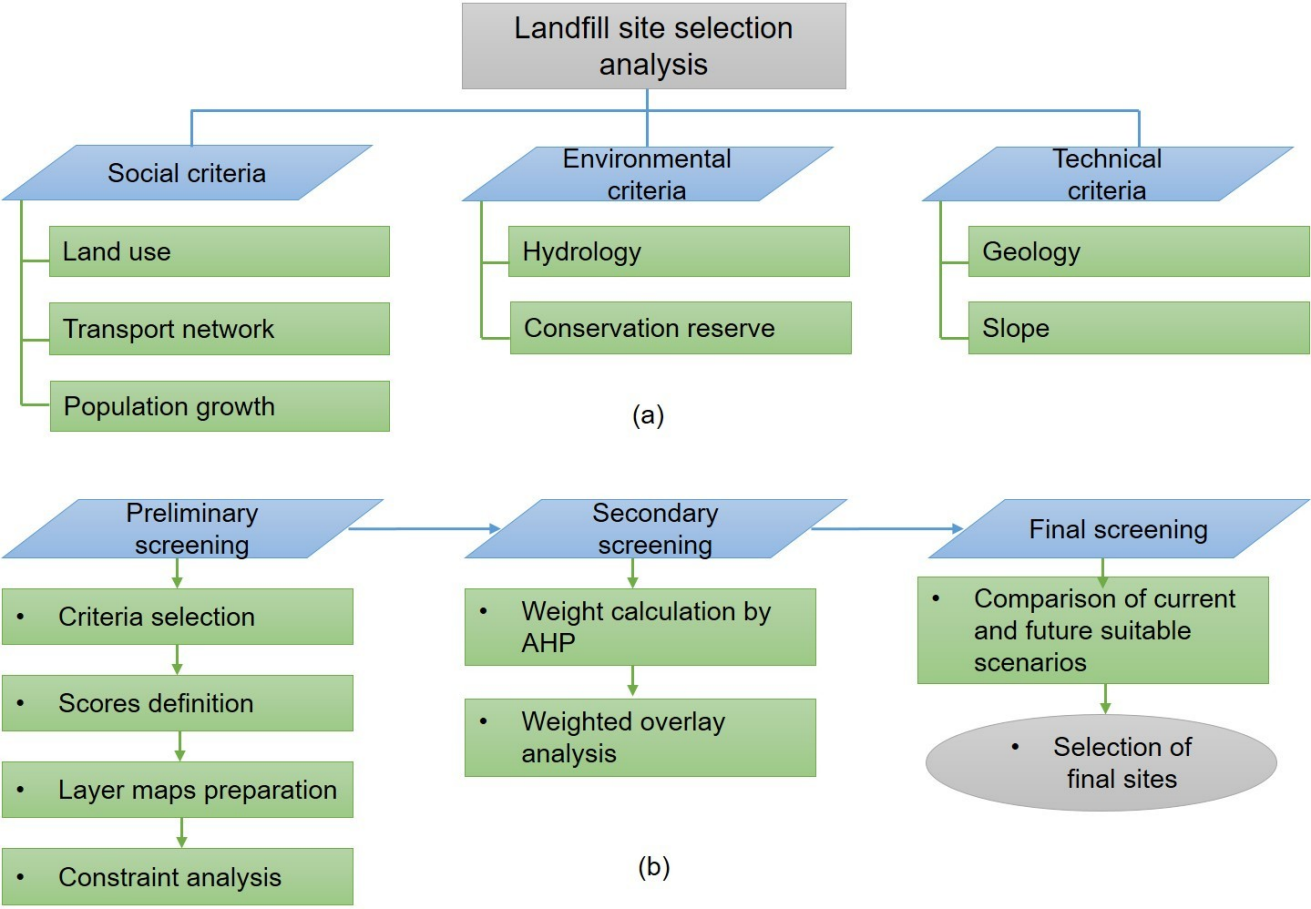
Criteria and methodology. (a) site selection criteria; (b) steps for assessing landfill site suitability.

The study consisted of three main phases: i) preliminary screening based on the analysis of individual criteria maps; ii) weighted overlay of suitable scenario based on the AHP; iii) analysis of the population projection and identification of optimal areas (Fig 3).

Based on diverse scientific articles [9,15,31] national waste management guidelines [32] and international landfill guidelines [33], six criteria that belong to social, technical and environmental categories were selected in this study, namely: land use, transport network, geology bedrock, slope, hydrology and distribution of conservation reserves [13,31–33].

ArcGIS 10.5.1 (ESRI, Japan) was used for the study. The criteria data were obtained as vector files at the National Centre of Cartography and Remote Sensing of Mozambique and later converted into raster datasets. Three suitability classes were generated to group all different criteria into a common unit of analysis [9,31]. Three discrete values: 0, 1 and 2 were used to code all features and group them into a single scale of measurement (Table 1), where 0 represents *unsuitable*, 1 represents *moderately suitable* and 2 represents *highly suitable*. The criteria were reclassified, weighted based on the AHP methodology, and overlaid using the spatial analysis tool of the ArcMap environment.

**Table 1:** Suitability criteria and scores.

Category	Criteria	Attribute	Score (unitless)
Social	Land use	Irrigated agriculture, industrial areas, crop production, mangrove	0
		Dryland agriculture	1
		Shrubland, grassland	2
	Transport networks	< 100 m	0
		> 3000 m	1
		100–3000 m	2
Technical	Geology	Carbonate rock, alluvium	0
		Terrestrial sediment, aeolian sand, red sandstone	1
		Igneous rock, volcanic arenite and clay	2
	Slope	> 15%	0
		0–3%	1
		3–15%	2
Environmental	Hydrology	< 500 m	0
		500–1500 m	1
		> 1500 m	2
	Conservation reserve	< 1000 m	0
		1000–3000 m	1
		> 3000 m	2

### Criteria of suitability and data preparation

#### Land use

About 48% of the greater Maputo region comprises urban and semi-urban areas, industrial areas, irrigated and dryland agriculture areas, all concentrated in the center and progressively expanding towards the west and north of the region; mangrove forests and floodplains are located in the coastal areas. The main land cover types in the areas surrounding the greater Maputo area were shrublands and grasslands. Peripheral areas are preferable for siting landfills because they are relatively close to urban areas and can therefore reduce transportation costs, but they are sufficiently far away to reduce adverse effects on human health. These land uses were categorized into three suitability categories (Table 1), converted to raster format, and reclassified. The land use map was obtained from the National Center for Cartography and Remote Sensing of Mozambique (based on Landsat SRTM, 2014).

#### Transport networks

Peripheral areas are preferable for siting landfills because they are relatively close to urban areas and can therefore reduce transportation costs, but they should be sufficiently far away to reduce adverse effects on human health [13,15,31]. The disposal facilities at operation in Maputo and Matola cities are located at distances less than 50 m away from roads, which may cause air quality issues and deterioration of landscape. Therefore, 100 m is a minimum buffer distance to protect the citizen’s health, landscape while maintaining affordable transportation costs. In spite of the buffer distance, proper engineering and sanitary measures are still necessary and must be included in the construction of new disposal facilities [31]. Additionally, the expansion of Maputo city from the center, to its outskirts, in the past 20 years has occupied a new area of approximately 5000 m. Thus, the distances between 100 m to 3000 were the considered acceptable, based on past trend of land occupation. Multiple buffer zones were created around roads (Table 1), and these were reclassified and converted to raster format. Due to the poor quality of smaller transport networks, only major roads, national and municipal networks were considered in this study.; these were obtained from the National Centre for Cartography and Remote Sensing of Mozambique.

#### Geology

The greater Maputo area consists of Cretaceous rocks, quartzitic sandstones and dark argillite, overlaid by limestone, clay marls and sandstones [34,35]. The bedrock geology was considered to be a technical criterion because it can be used for preliminary assessments of the underlying stability of a potential landfill [33,36]. For instance, clay soils can act as an effective liner system and so can reduce the construction costs of landfills. Furthermore, clay soils are not very permeable and can therefore be used to protect groundwater resources [36]. The coastal areas are covered by aeolian sands while the rivers are surrounded by gravels and clays. The majority of the area is covered by sandy soils while the western areas are comprised of clay soils [35,37].

#### Slope

The greater Maputo area is located predominantly on a plain with some sand dunes dating from the Quaternary Period [34]. The slope of the area is thus generally flat and gentle with the average elevation of below 100 m [35]. The slope of an area is also a technical criterion that affects landfill stability and maintenance. It can affect the drainage, compromise surface and groundwater [11]. Hence, gentle slopes facilitate leachate circulation and maintenance, whereas slopes that are either too flat or too steep make leachate collection difficult and can also increase construction costs [22]. Therefore, the area was reclassified into three slope classes, as shown in Table 1. The slope map was extracted from the digital elevation model (DEM) of the study area, downloaded from the United States Geological Survey (USGS) database that was compiled using the USGS Earth Explorer SRTM 1 data, with 1 arc second and a cell size of approximately 30 m per cell.

#### Hydrology

It is important that potential landfills are placed at some distance from rivers for two major reasons; first, to protect the water bodies and related ecosystems from contamination and deterioration, second, to protect landfills physical integrity and maintenance costs [11,31,32,38]. According to the [39] along with research articles which suggest the minimum buffer distances between water bodies and streams and other developments, multiple buffer zones were created around the three major rivers in greater Maputo region in this study. These distance ranges are shown in Table 1. The hydrology was extracted from the DEM of the study area.

#### Conservation reserve

In the greater Maputo region, a conservation reserve was designed to protect special fauna [37] and must be protected from contamination by a potential waste disposal site [31,32,39]. The nature reserve information was obtained as a layer map produced by the National Centre for Cartography of Mozambique. As before, buffer zones were created and these were reclassified based on suitability scores (Table 1).

### Analytic hierarchy process (AHP) and criteria weighting

The AHP is commonly applied to problems associated with potentially conflicting decisions due to its many advantages such as user-friendliness, transparency and capacity to integrate heterogeneous data [40–42]. In this study, the AHP was used as a support tool to calculate the criteria weights, as opposed to assigning them carelessly. Furthermore, this statistical method was used as an instrument to harness the perception of the real issues that waste planners consider relevant, therefore, require priority in the current waste management system.

The AHP was conducted based on the input of experts collected through forms. The criteria weights were calculated based on a comparison matrix where all the attributes were compared against each other, based on a nine-point scale of relative importance [11,39]. The next step consisted of normalizing the sum of the values in each column and calculating the Consistency Ratio (CR) to verify if the values were assigned randomly or not; hence, if the comparisons were done in a consistent manner, the CR should be lower than the value 0.1. The equation for this calculation is presented below:

CR=CI/RI (1)

where, CR is the consistency ratio, RI is the Random Index (with predefined fixed values), and CI is the Consistency Index. The consistency index was calculated using Eq 2:

$$CI=\frac{\lambda \max-n}{n-1}$$ (2)

where *λ*_*max*_ is the maximum eigenvalue of the pairwise matrix and *n* is the number of variables being calculated.

### Population projection

The population projection plays a significant part in planning future infrastructure [43,44] and was regarded as an indicator of how the municipalities will grow, occupy more space, and produce more waste. Thus, waste facilities need to be planned in advance, based on population projection for the greater Maputo area, to effectively prevent social and environmental disturbances.

The population projection data was collected at the National Institute of Statistics of Mozambique [30]. The estimation of population until the year 2037 was based on the arithmetic projection method, which is useful if considering the population growth at a constant rate at a relatively short time (Eq 1, based on [44]). The population density of the year 2017 was equally calculated and compared to that of 2037 to estimate the evolution of potential waste disposal scenarios. The formula is shown below:

$$P_{t}=P_{0}+K_{a}(t–t_{0})$$ (3)

Where P_t_ = population at some time in future, P_0_ = present or initial population; K_a_ = population growth rate; t = data in which the population is estimated and to = date of the first available census. The K_a_ coefficient was calculated based on the formula below:

$$Ka=\frac{Pn-Po}{tn-to}$$ (4)

Where P_n_ is the population in the last year of the census; P_o_ is the population in the first year; t_n_ is the last year of the census and t_o_ is the year of first census.

The results of the projection were converted into input data to create population density maps for the greater Maputo area, based on the following scores: *0–3000 inhabitants/Km*^*2*^ *(highly suitable)*; 3*000–6000 inhabitants/Km*^*2*^ *(moderately suitable); greater than 6000 inhabitants/Km*^*2*^ *(unsuitable)*. These maps indicate that the higher the population density, the lesser the space available for new waste facility constructions; and were overlaid the previously constructed criteria map, based on social, environmental and technical criteria.

## Results and discussion

### Reclassification and constraint analysis

**Fig 4:**
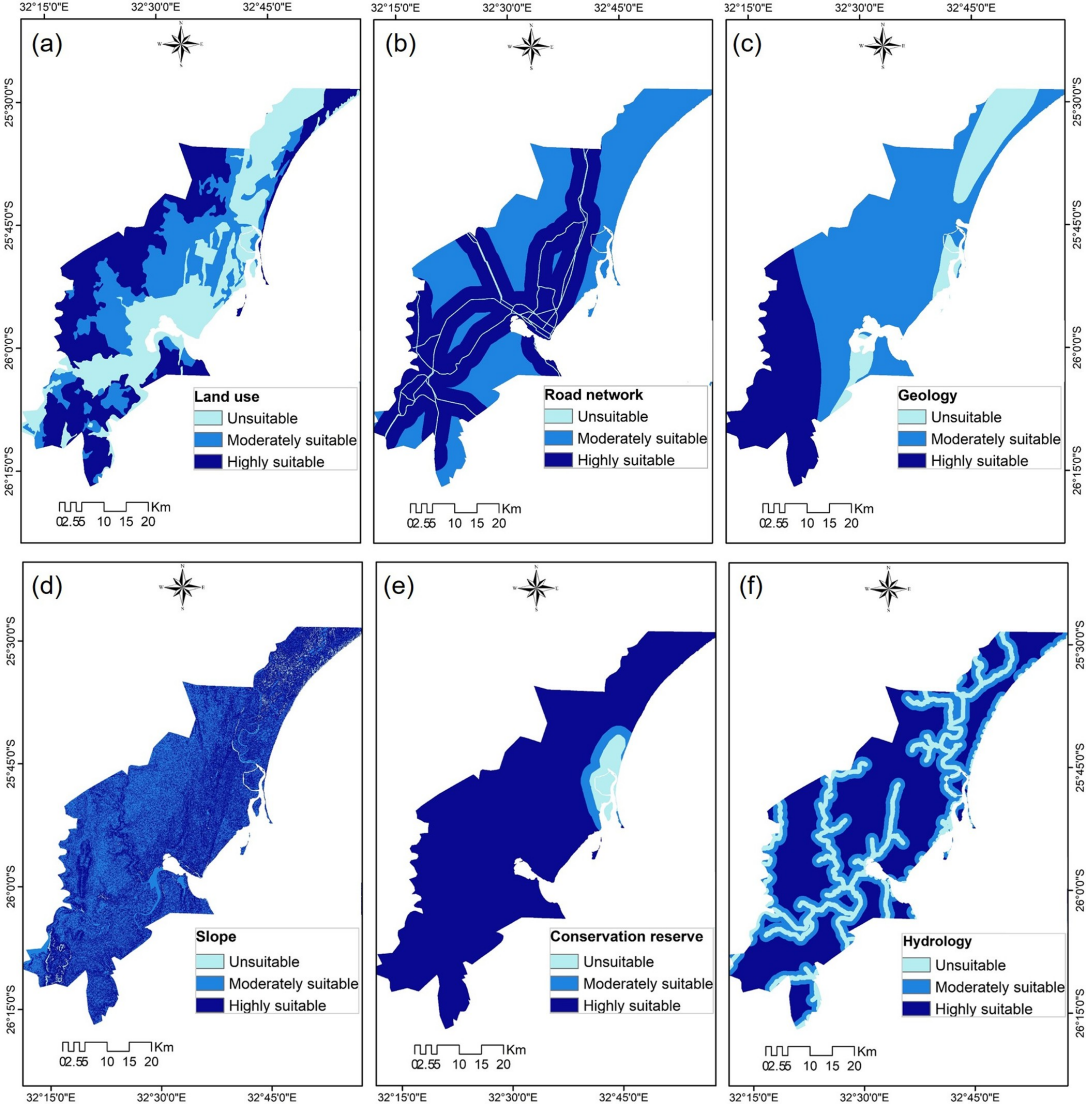
Criteria maps. Classification of unsuitable, moderately suitable and highly suitable areas: (a) land use; (b) transport network; (c) geology; (d) slope; (e) conservation reserve; (f) hydrology. Maps were edited from Landsat [45] and USGS EROS [46].

The constraint analysis consisted of removing locations that could cause environmental damage and human health problems as result of landfill construction. The results are shown in Fig 4, where the areas colored light blue indicate the location of least suitable areas; while remaining areas, shown in blue and dark blue, show the location of moderately suitable and highly suitable areas, respectively.

#### Social criteria

In the land use map, the central and coastal areas, representing urban features and other land uses are not considered suitable for use as landfills, whereas areas to the west and northwest are moderately and highly suitable, respectively (Fig 4A).

In the map showing the transport networks (Fig 4B), the buffer zones created around roads reveal that the central and southern areas are highly suitable, whereas the areas to the west and northwest were moderately suitable. Because urban areas tend to expand along transport ways, transport routes match with the major urban area of the land use map [6]. However, due to the buffer zones surrounding these transport routes, the degrees of suitability of these two social variables (land use and transport network) differ in terms of their distribution.

#### Technical criteria

The results obtained after reclassifying the technical criteria revealed that the study area was generally well suited for the establishment of landfills (Fig 4C and 4D). The most optimal sites were located to the west due to the natural occurrence of igneous rock formations and clay soils with gentle slopes. The coastal areas, although had a gentle slope, are unsuitable for the establishment of landfills because they comprise alluvium and limestone [36,47]. Moderately suitable areas could be identified in both maps. In the geology map (Fig 4C), the remaining areas, comprised of sandstone formations, are considered moderately suitable because they require the application of more sophisticated engineering methods that could be costly. In the slope map (Fig 4D), moderately suitable locations were identified towards the west, and there were very few if any unsuitable areas.

#### Environmental criteria

River water protection (hydrology map) was accomplished by generating buffer zones around rivers, which left the remaining areas of the map free for any required developments (Fig 4F). This step was in accordance with the necessity of establishing distances to protect water resources recommended by [31] and other studies [11]. The existing nature reserve park (Fig 4E) was also successfully protected by a minimum buffer zone of 1000 m, which was in accordance with [31]. Consequently, most of the remaining areas of the map were suitable for landfill development.

### Weighted overlay map

The attribute weights which result from the pairwise comparison is shown in Table 2; and were used to overlay the final map, prior to the inclusion of the arithmetic growth projection data. The land-use layer map was the most significant, consequently has greatly influenced the final weighted overlay. This result indicates the preferences given to the social category by the stakeholders and may be explained by the fact that currently, in the Greater Maputo area, land-use conflicts are the major hindrance for the advance of the regional landfill, previously selected to respond to citizens of both Maputo and Matola cities.

**Table 2:** Pairwise comparison of criteria giving a higher weighting to land use.

Criteria	LU	TN	GEO	SLP	HYDRO	CR	Criteria Weight
LU	1	2	6	6	2	3	34.5
TN	1/2	1	5	5	2	3	25.8
GEO	1/6	1/5	1	1	1/4	1/4	4.6
SLP	1/6	1/5	1	1	1/4	1/4	4.6
HYDRO	1/2	1/2	4	4	1	2	17.7
CR	1/3	1/3	4	4	1/2	1	12.9

λ_max_ = 6.1619, RI = 1.24, CI = 0.03238, CR = 0.0261 <1.0.

LU–land use; TN–transport network; GEO–geology; SLP–slope; Hydro–hydrology; CR–conservation reserve.

Unsuitable areas occupy about 48% of the map, and match with urban areas distributed along the coast, center, north and parts of the south. Moderately suitable areas occupy about 19%, distributed in the center of the greater Maputo area and coincide with dryland agriculture. Highly suitable areas occupy about 33% of the map and are consistent with grasslands and shrublands.

### Population growth projection and municipal solid waste management

**Fig 5:**
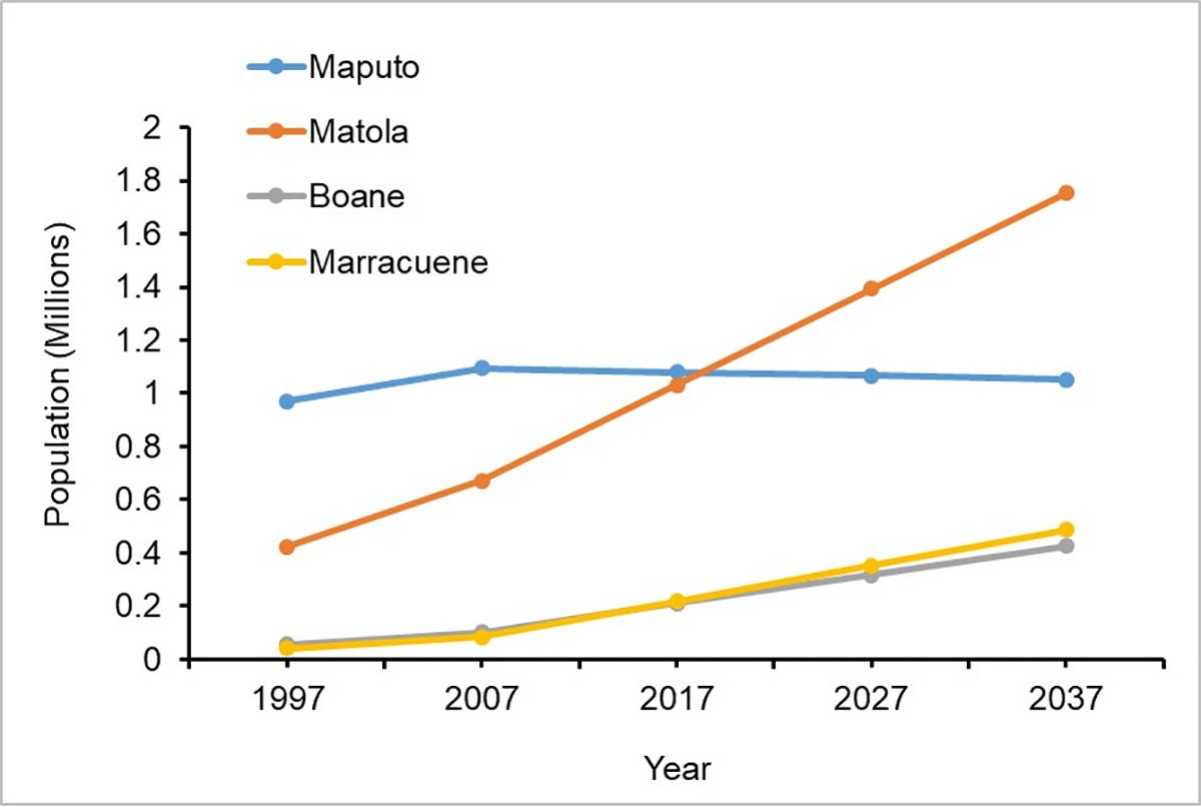
Past and future population growth, from 1997 to 2037.

**Fig 6:**
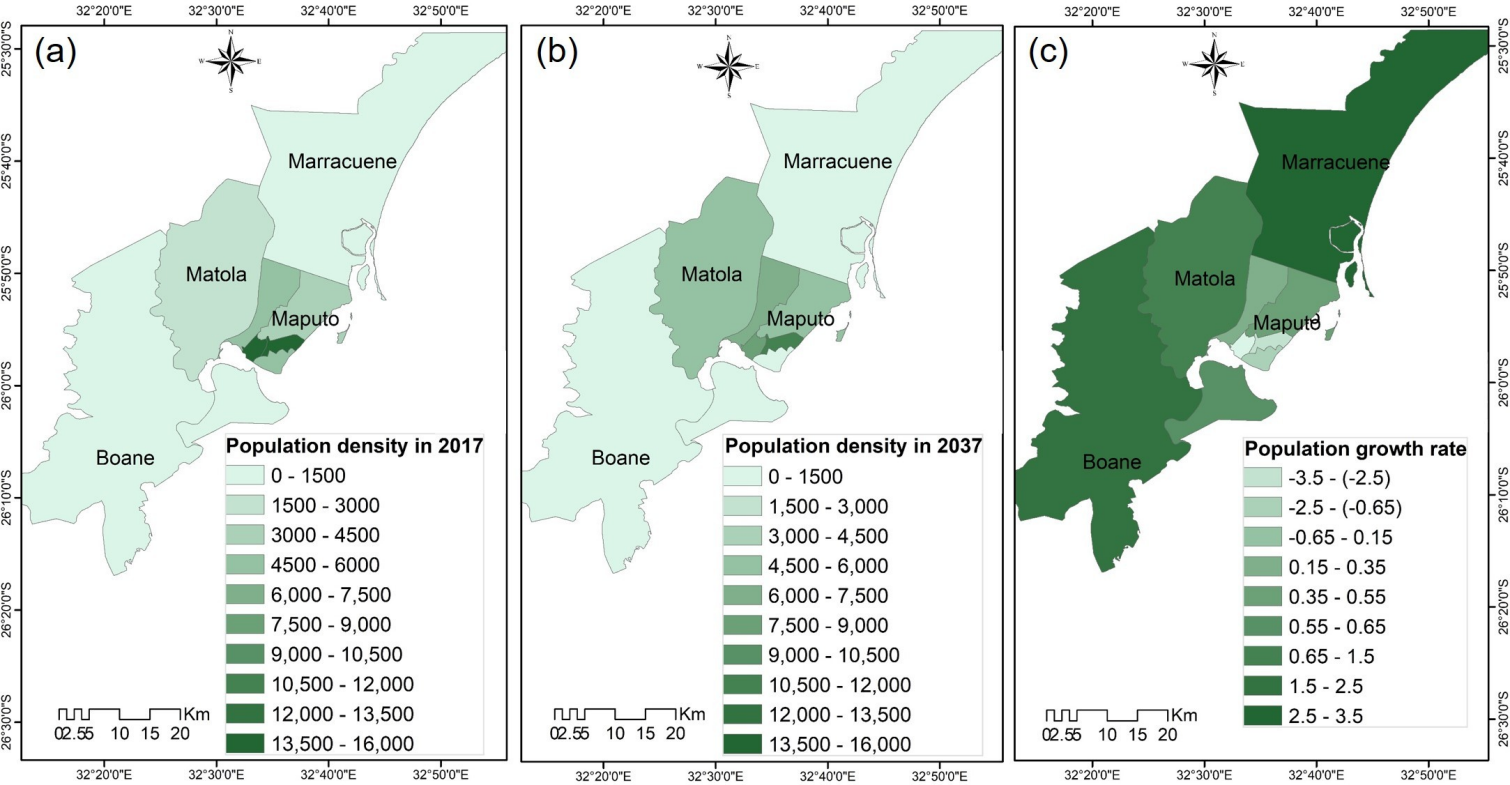
Population projection scenarios. (a) population density in 2017 (inhabitants/Km^2^); (b) population density in 2037 (inhabitants/Km^2^); (c) population growth rate average (%). Maps were edited from the Landsat [45] and USGS EROS [46].

Results of arithmetic projection of the population in the greater Maputo area indicate that the total population will increase to approximately 4 million people in 2037, almost doubled compared to the year of 2007 (Fig 5), and the population growth in the 4 cities has been occurring at different rates. Maputo city had approximately 54% of the population of the great Maputo area by the year 2007, but this number is expected to reduce to 28% around the year 2037. Maputo city had an average population density closer to 3,400 inhabitants/Km^2^ in the year 2017 (Fig 6A), with two of its central boroughs reaching the highest population densities of 14,000 and 16,000 inhabitants/Km^2^, respectively. The population density is supposed to only slightly decrease to approximately 3000 inhabitants/Km^2^ in 2037 (Fig 6B). The population growth rate map (Fig 6C) indicates that the population growth rate in Maputo city, especially in the central boroughs have decreased, while it has increased in Marracune, Matola and Boane cities. This is a result of the high population density of Maputo city which has caused the outward expansion away from the main built-up area, to neighboring cities.

Matola city has been particularly affected by this growth but is expanding in a pattern of uncontrolled urban sprawl due to inadequate institutional coordination and poor space planning. Matola city, which in 2017 had a population density close to 3,000 inhabitants/Km^2^, will grow to approximately 5,000 inhabitants/Km^2^ in 2037 (Fig 6). Marracuene municipality had a population density of only 320 inhabitants/Km^2^ but is likely to reach about 700 inhabitants/Km^2^ in 2037. Finally, the least populated municipality of Boane, with a population density of approximately 270 inhabitants/Km^2^, is expected to reach only about 500 inhabitants/Km^2^ in 2037.

One result of this rapid expansion is that MSWM facilities could not improve at same rate as the increase in the demand for waste collection and disposal [5]. Currently, approximately 2,700 tons of MSW are generated per day in the whole area (Table 3), comprising mostly organic matter, and disposed of in two landfills [48].

**Table 3:** Current and future projected waste generation in the greater Maputo area, by city.

City	Year 2015		Year 2037	
	Population	Waste (ton/day)	Population	Waste (ton/day)
Boane	188,805	203	425,991	458
Matola	960,069	1,032	1,753,479	1,885
Marracuene	192,025	206	486,414	523
Maputo	1,083,147	1,164	1,051,575	1,130

The first municipal landfill in Maputo (Fig 2A), which was constructed about 50 years ago has reached its peak [48]. The second landfill site that was constructed in Matola (Fig 2B), which is in an inappropriate location, close to a river, housing and industrial areas [49]. Neither of the landfill sites currently comply with the environmental regulations of [31] due to their proximity to housing areas and lack of leachate and gas control measures [5,32,33,38]. Consequently, another regional landfill site was planned for development in Matola city to replace the existing sites (Fig 2C). However, due to the continuous uncontrolled expansion of the city and conflicts with citizens in the vicinity, the landfill placement is still a problem [48,49].

According to the predicted population growth, MSW generation will increase from the current 2,700 tons per day to approximately 4,000 tons per day in the 2037 scenario. Maputo city which in 2015 produced approximately 45% the MSW, will reduce to 28.7% in 2037. On the contrary, Matola city produced 40% of MSW in 2015 but will expand to 47% in 2037. Marracuene and Boane municipalities will increase the MSW generation from the 8% in 2015 to 13% and 11% in 2037, respectively.

The past population growth in the greater Maputo area has led to a burden in the MSW and overuse of disposal facilities [5,25]. Maputo city has already experienced the effects of this growth in the excessive usage of waste disposal facilities but is now registering a decrease in its MSW generation in parallel with the population decrease. Maputo city is not recommended for placement of new landfills. On the other side, Matola city continues to experience the population growth and can be considered for landfill placement. A landfill in this city would be the most recommended because it can serve all other municipalities; however, a proactive assessment should be done by all municipalities in combination. On the contrary, Boane, and Marracuene municipalities, both with a population density lower than 1000 inhabitants/Km^2^, deal with great distances between settlements and waste generation below 1 kg/person/day. This scenario is typical of areas transitioning from rural to urban, thus, waste elimination methods need to be adjusted to these types and quantity of waste [16]. Therefore, these two municipalities could benefit from solutions such as smaller composting facilities and transfer stations that would connect the waste to Matola city landfill. The future scenario indicates that a higher population growth will need the provision of collection, treatment and disposal of waste.

### Selection of optimal landfill sites

**Fig 7:**
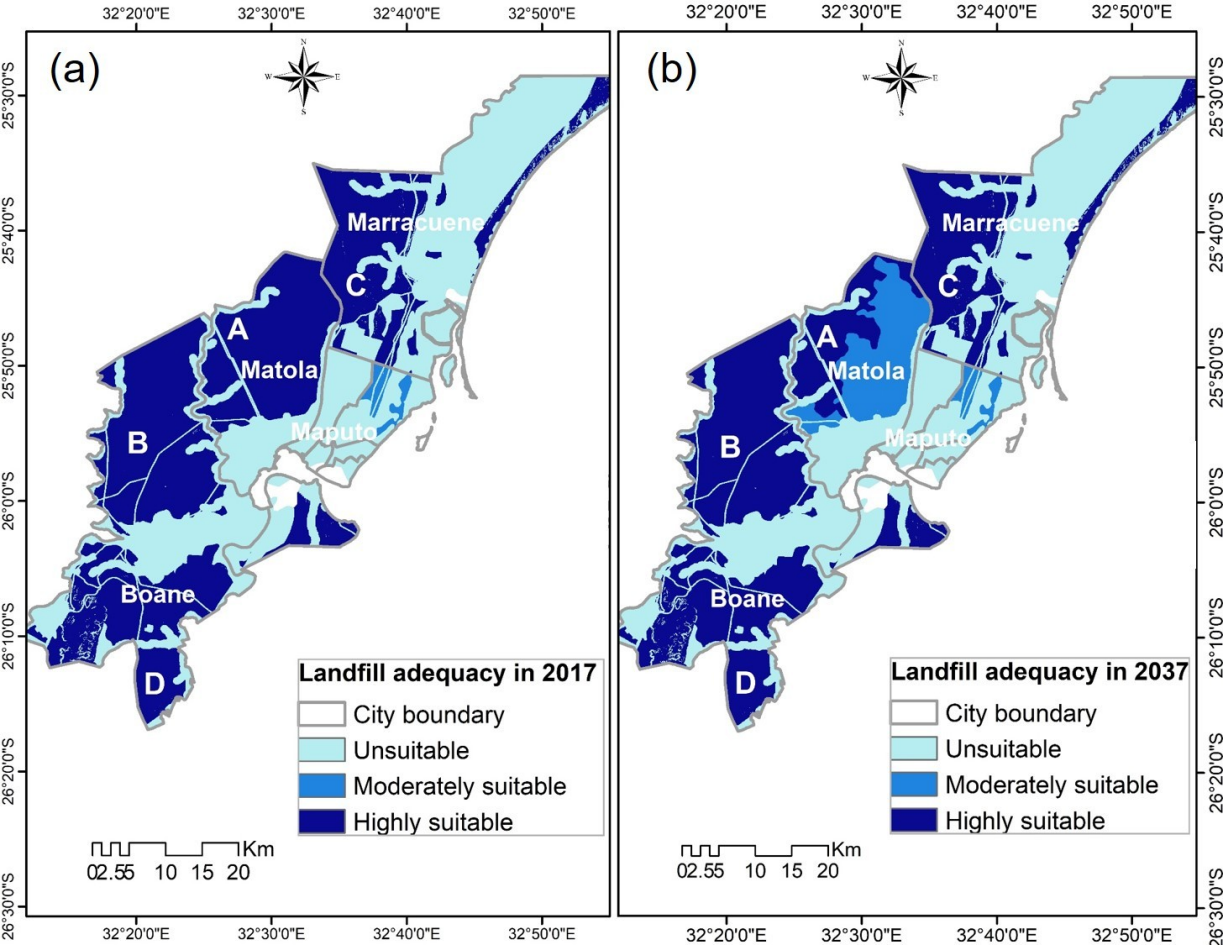
Final suitable maps. (a) Scenario 1 –weighted overlay for 2017; (b) Scenario 2 –weighted overlay for 2037. Maps were edited from the Landsat [45] and USGS EROS [46].

The population density projection combined with the analysis of [32,33] were considered to determine the social, environmental, and technical suitability for effective landfill placement. Fig 7 shows the results of the weighted overlay of all criteria maps with the population density maps. The area was divided into three classes: unsuitable, moderately suitable, and highly suitable, which are shown as light to dark blue in the map, respectively. The land use map was determinant in the final scenario maps. For instance, bushland and grassland areas were considered very suitable, areas of dryland agriculture were considered moderately suitable, and urban areas and irrigated agricultural areas were unsuitable.

Fig 7A shows the first adequacy scenario, considering only the population density in the year 2017. It shows 48% of the area as unsuitable for landfill placement, 2% of the areas as moderately suitable, and 50% of areas as highly suitable.

In this scenario map, Maputo city is unsuitable due to the combination of alluvium soils and urban areas with high population densities. Marracuene municipality is unsuitable in east areas, due to the presence of alluvium soils, water streams, and agriculture fields, but is suitable in west areas due to very low population density and sandy soils. Matola city is highly suitable in west parts due to the moderate population density; but unsuitable in the central parts due to concentration of residential and industrial areas. Boane municipality is also unsuitable in the center due to the presence of settlements and irrigated agriculture fields; but is highly suitable in the south and west due very low population density combined with clayey soils, which are the preferred cover materials for landfills.

The adequacy scenario (scenario 2) considering the population density in the year 2037 is shown in Fig 7B. This scenario conforms with the assumption that the population expansion from Maputo city would lead to an increase in population in Matola city and, consequent reduction in land availability [50]. This scenario shows 49% of the areas are unsuitable for landfill placement, 9% of the areas are moderately suitable, and 43% of the areas are highly suitable. The increase of population may result in lesser highly suitable areas for landfill. Scenario 2 (Fig 7B) also suggests that the pronounced changes in population density in Matola city may significantly change the landfill placement decision making, since the areas that were highly suitable in scenario 1 (Fig 7A) have reduced and became moderately suitable in scenario 2 (Fig 7B).

It is possible to see a correlation between population density and space decrease, however, it becomes more or less evident based on the values defined as acceptable or high for population density, which must be adjusted to the study area. Nonetheless, due to the dynamic nature of population evolution, this is a variable that can alter previously fixed waste management plans.

In both Scenario 1 and 2 (Fig 7), areas A, B, C, and D are equally adequate for landfill placement. Area A in Matola cities, however, is the most recommendable of all because it can serve both Maputo and Matola city, simultaneously. Moreover, landfills in area A can still comply with environmental regulations while having affordable costs.

The spatial analysis carried in this study is critical to identifying optimal landfill sites, but it needs to be supported with on-site studies. Thus, a future research direction is to perform risk assessments of chemical pollution associated with landfill sites because such assessments can assist the improvement of standards of soil and groundwater protection.

## Conclusions

In this study, landfill placement in the greater Maputo region was examined using a multi-criteria decision-making approach, which combines the processes of preliminary screening, generating, and superimposing layer maps and examining the present and future population growth. The approach considered social, environmental and technical requirements criteria (i.e., land use, transport networks, hydrology, conservation reserve, geology and slope); all of them were overlaid in the GIS using an AHP.

The arithmetic projection of population trend shows that the great Maputo area is experiencing a rapid and uncontrolled population growth. Especially, the population density of Matola city is expected to grow from 3,000 inhabitants/Km^2^ at present to 5,000 inhabitants/Km^2^ in 2037. Based on the current population (year of 2017) and projected population in (year of 2037), combined with multi-criteria, two scenarios were created. The scenario considering future population shows less highly suitable (43%) areas for landfills than the one considering current the population (50%), suggesting that the pronounced changes in population density significantly affect the landfill placement decision making. Finally, the western part of Matola city is recognized as the most recommendable landfill site, which can serve both Maputo and Matola city with affordable costs.

The approach presented in this study newly includes an analysis of population growth, which can be used not only to visualize city expansion tendencies but also to landfill placement decision making in any area.

## Supporting information

S1 Fig Weighted overlay of six criteria. (TIF)
