# Supplementary figures and images for: Dynamic evaluation method for planning sustainable landfills using GIS and multi-criteria in areas of urban sprawl with land-use conflicts
Source: PLoS One. 2021 Aug 27;16(8):e0254441. doi: 10.1371/journal.pone.0254441 (PMC8396732; doi:10.1371/journal.pone.0254441)

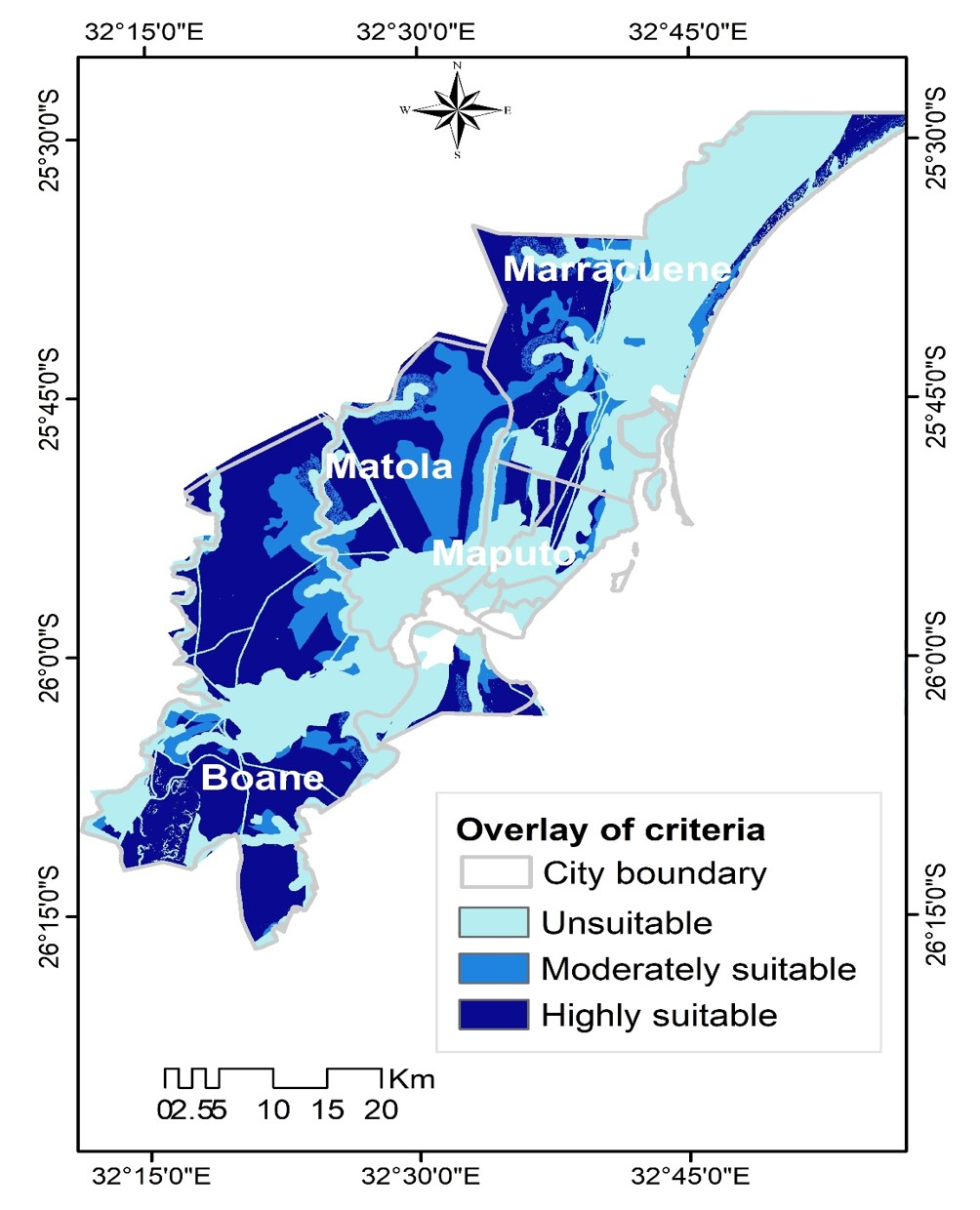

Supplement: S1 Fig — (TIF) [file pone.0254441.s001.tif]
